# SIRT1 at the crossroads of AKT1 and ERβ in malignant pleural mesothelioma cells

**DOI:** 10.18632/oncotarget.7321

**Published:** 2016-02-11

**Authors:** Giulia Pinton, Sara Zonca, Arcangela G. Manente, Maria Cavaletto, Ester Borroni, Antonio Daga, Puthen V. Jithesh, Dean Fennell, Stefan Nilsson, Laura Moro

**Affiliations:** ^1^ Department of Pharmaceutical Sciences, University of Piemonte Orientale “A. Avogadro”, 28100 Novara, Italy; ^2^ Department of Sciences and Technological Innovation, University of Piemonte Orientale “A. Avogadro”, 15121 Alessandria, Italy; ^3^ Department of Health Sciences, University of Piemonte Orientale “A. Avogadro”, 28100 Novara, Italy; ^4^ Department of Integrated Oncological Therapies, IRCCS San Martino-IST, 16132 Genova, Italy; ^5^ Division of Biomedical Informatics Research, Sidra Medical and Research Center, 26999 Doha, Qatar; ^6^ Department of Cancer Studies, Cancer Research UK Leicester Centre, University of Leicester, LE1 7RH Leicester, UK; ^7^ Department of Biosciences and Nutrition, Karolinska Institutet, S-141 57 Huddinge, Sweden; ^8^ Karo Bio AB, Novum, S-141 57 Huddinge, Sweden

**Keywords:** malignant pleural mesothelioma, AKT1, SIRT1, estrogen receptor beta

## Abstract

In this report, we show that malignant pleural mesothelioma (MPM) patients whose tumors express high levels of *AKT1* exhibit a significantly worse prognosis, whereas no significant correlation with *AKT3* expression is observed. We provide data that establish a phosphorylation independent role of AKT1 in affecting MPM cell shape and anchorage independent cell growth *in vitro* and highlight the AKT1 isoform-specific nature of these effects.

We describe that AKT1 activity is inhibited by the loss of SIRT1-mediated deacetylation and identify, by mass spectrometry, 11 unique proteins that interact with acetylated AKT1.

Our data demonstrate a role of the AKT1/SIRT1/FOXM1 axis in the expression of the tumor suppressor ERβ. We further demonstrate an inhibitory feedback loop by ERβ, activated by the selective agonist KB9520, on this axis both *in vitro* and *in vivo*.

Our data broaden the current knowledge of ERβ and AKT isoform-specific functions that could be valuable in the design of novel and effective therapeutic strategies for MPM.

## INTRODUCTION

Human malignant pleural mesothelioma (MPM) is an aggressive cancer, closely linked to asbestos exposure, with very poor survival rates [1, 2]. Surgery in combination with radiation and chemotherapy is used for patients with early stage disease, but most patients have unresectable disease and are treated mainly with palliative chemotherapy [3, 4]. In a first line setting, pemetrexed in combination with cisplatin has been accepted as an almost universal standard [5]. In the second line setting, various chemotherapy agents are used, either as monotherapy or as part of polytherapy, but none has been validated and no approved drugs reverse disease progression [6, 7].

The PI3K/AKT signaling pathway is aberrantly active and has an important biologic impact in MPM progression and chemo-resistance [8, 9]. AKT serine-threonine kinases function as critical regulators of tumor cell survival, proliferation, metabolism and migration. Moreover, enhanced AKT activity confers resistance to endocrine and molecular-targeted therapeutics including cytotoxic and genotoxic drugs. Three isoforms of AKT have been identified in mammals: AKT1, AKT2 and AKT3 [10]. All AKT isoforms possess *in vitro* transformation ability [11]. However, there may be isoform-specific functions in tumor cells due to amplifications and mutations of upstream components of the PI3K/AKT signaling pathway [12]. Findings from AKT isoform-specific knockout mice suggest that the functions of the different AKT kinases are not completely overlapping and that isoform-specific signaling contributes to the diversity of AKT activities [13]. AKT is generally activated in a multistep process that includes (i) binding to phosphatidylinositol 3,4,5-trisphosphate (PIP3), (ii) translocation from the cytosol to the membrane, and (iii) phosphorylation at Thr308 and Ser473 by the upstream kinases PDK1 (phosphoinositide-dependent protein kinase 1) and mTORC2 (mammalian target of rapamycin (mTOR) complex 2) [14–16]. Reversible acetylation of lysine residues, by histone acetyltransferases (HATs) and histone deacetylases (HDACs), was recently described as a post-translational regulatory mechanism that controls the activity of AKT [17].

SIRT1, a prototypical member of the class III HDACs collectively called sirtuins [18, 19], deacetylates the PH domain of AKT, a process that is necessary for AKT binding to PIP3, membrane localization and activation [17]. Despite it has been described that induction of SIRT1 by caloric restriction reduces cell proliferation and tumor formation in a mouse model of colon cancer [20], SIRT1 abundance is increased in various types of tumors [21]. These tumors also show increased activation of AKT suggesting that SIRT1 might promote cancer by activating AKT [22, 23].

Recent research shows that the forkhead transcription factor FOXM1, a downstream effector of the PI3K/AKT/FOXO signaling pathway is overexpressed in MPM [24]. Connections between FOXM1 and SIRT1 have been recently described in gliomas [25]. FOXM1 has pivotal roles in tumorigenesis and in chemotherapy sensitivity [26]. Moreover, FOXM1 is linked to the induction of epithelial–mesenchymal transition (EMT), a process that renders tumor cells more invasive and aggressive [27].

Our group previously published that MPM derived cell lines express both AKT1 and -3 isoforms [28]. We recently described that AKT1 is involved in the regulation of ERβ expression, a tumor suppressor and positive prognostic factor in patients diagnosed with MPM [29]. Moreover, we reported that ERβ, activated by the selective agonist KB9520, significantly inhibited AKT phosphorylation/activation both *in vitro* and *in vivo* [30]. Consistent with AKT decreased phosphorylation we observed an increase in its acetylation due to inhibition of SIRT1 expression. Here, we further characterize the phosphorylation-independent functions of AKT1 in MPM cells and describe the role of SIRT1 in the cross-talk between AKT1 and ERβ.

## RESULTS

### Expression of AKT1 but not of AKT3 negatively correlates with MPM patients' survival

**Figure 1:**
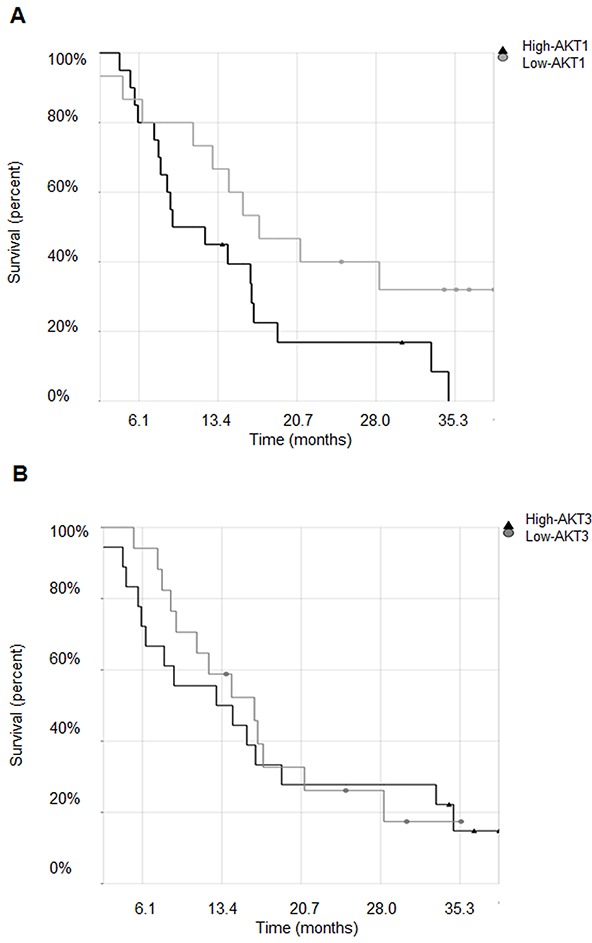
Expression of AKT1 but not of AKT3 negatively correlates with MPM patients' survival **A.** Kaplan-Meier survival curves of malignant pleural mesothelioma patients stratified for *AKT1* and **B.** *AKT3* high or low expression levels.

We performed *in silico* analysis of microarray and clinical data to correlate *AKT1, AKT2* and *AKT3* expression to MPM patients' survival. Raw data from the publicly available MPM microarray gene-expression data set (n=40), GSE 2549, was pre-processed and normalized using the Robust Multichip Average method. The probe for *AKT2* was removed when the dataset was filtered using the Affymetrix ‘Absent’ flag call, showing lack of expression. The median expression level for the probe set was used to stratify the patients in groups with high or low *AKT1* and *-3* expression levels, respectively, and Kaplan-Meier survival analysis was performed. As shown in Figure 1A, patients whose tumors expressed high levels of *AKT1* exhibited a significantly worse probability of survival (p=0.05), while no significant correlation (p=0.75) with *AKT3* expression was observed (Figure 1B). Furthermore, no significant association was found between *AKT1* or *AKT3* levels and histologic subtypes (p=0.4, p=0.89, respectively).

### AKT1 silencing affects MPM cell morphology and anchorage-independent growth

**Figure 2:**
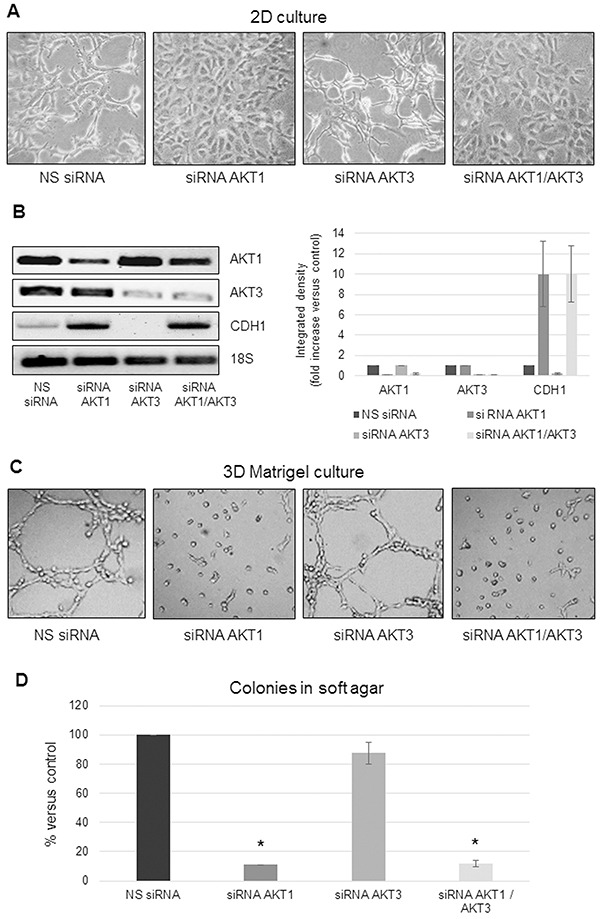
AKT1 silencing affects MPM cell morphology and anchorage-independent growth **A.** Phase contrast images (200X magnification) of MSTO-211H cells transfected with non-specific control siRNA (NS siRNA) or *AKT1* siRNA (siRNA AKT1), *AKT3* siRNA (siRNA AKT3) or both specific siRNAs (siRNA AKT1/AKT3). **B.** Representative RT-PCR analyses and relative densitometry of *AKT1*, *AKT3*, and *CDH1* in *AKT1* and *AKT3* silenced MSTO-211H cells compared to their controls. 18S rRNA was used as housekeeping gene. **C.** Phase contrast images (200X magnification) of MSTO-211H cells transfected with non-specific control siRNA or *AKT* isoform specific siRNAs grown on Matrigel coated dishes for 24 hours. **D.** Soft agar colony counts in non-specific control siRNA or *AKT* isoform specific siRNAs transfected MSTO-211H cells. Columns represent the percentage of the mean number of colonies versus control ± s.d.; * p≤0.05.

We recently described that decreased *AKT1* expression caused a dramatic change in MSTO-211H cell shape [30]. Here we show that knockdown of the *AKT1* isoform reverted the MSTO-211H spindle-shaped cell morphology to a more epithelioid like, whereas knockdown of *AKT3* exaggerated the spindle-shaped phenotype (Figure 2A). By *AKT1* and *-3* double silencing experiments, we demonstrate that the epithelioid phenotype, induced by *AKT1* down-regulation, was dominant over the effect of knocked down *AKT3* (Figure 2A). Consistently with the observed change in cell morphology, an increase in *CDH1* (E-Cadherin coding gene) expression was observed in *AKT1* silenced cells, independently from *AKT3* expressed levels (Figure 2B). We further examined the role of *AKT1* and *-3* in cell adhesion/spreading on Matrigel-coated plates. While control or *AKT3* silenced MSTO-211H cells formed complex meshes of 2-3 cells in thickness, *AKT1* silenced cells did not spread on Matrigel (Figure 2C).

Moreover, decreased *AKT1* expression significantly compromised the capability of MSTO-211H cells to form colonies in soft agar whereas knockdown of *AKT3* expression did not result in significant variation in the number of colonies formed compared to the control (Figure 2D). These results support a role for AKT1 isoform in promoting epithelial to mesenchymal transition (EMT) and invasiveness of MPM cells. To reinforce our data, we over-expressed *AKT1* by transient transfection of the epithelioid MPM derived REN cells. RT-PCR and Western blot shown in Supplementary Figures S1A and S1B confirm the induction of *AKT1* expression and phosphorylation in transfected cells. Transfected cells acquired a more spindle-like phenotype when cultured in monolayer (Supplementary Figure S1C), increased branching when plated on Matrigel (Supplementary Figure S1C) and formed more colonies in soft agar (Supplementary Figure S1D). In accordance with the phenotype, in *AKT1* transfected cells the expression of *CDH1* was decreased (Supplementary Figures S1A, S1B).

### Inhibition of AKT phosphorylation is not sufficient to affect MPM cell morphology and anchorage-independent growth

It has been reported that AKT is frequently activated in MPM specimens and cell lines.

**Figure 3:**
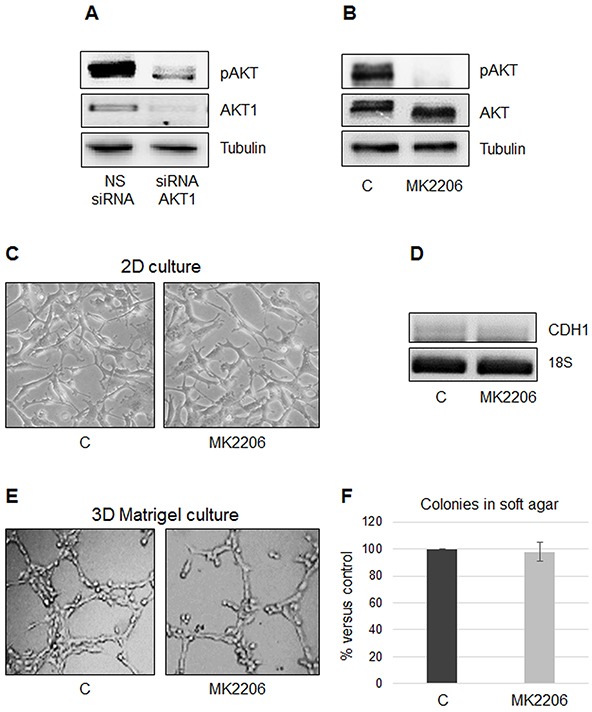
The specific AKT inhibitor MK2206 does not prevent growth on Matrigel or colony formation in soft agar **A.** Representative Western blot analyses of pAKT and AKT1 in MSTO-211H cells transfected with non-specific control siRNA (NS siRNA) and *AKT1* siRNA (siRNA AKT1). Tubulin was used as loading control. **B.** Representative Western blot analyses of pAKT and AKT in MSTO-211H cells untreated or treated for 24 hours with 8 nM MK2206. Tubulin was used as loading control. **C.** Phase contrast images (200X magnification) of MSTO-211H grown on untreated plastic for 24 hours ± 8 nM MK2206. **D.** Representative RT-PCR analyses of *CDH1* in MSTO-211H cells treated for 24 hours ± 8 nM MK2206. 18S rRNA was used as housekeeping gene. **E.** Phase contrast images (200X magnification) of MSTO-211H grown on Matrigel coated dishes for 24 hours ± 8 nM MK2206. **F.** Soft agar colony counts in MSTO-211H cells untreated or treated with 8 nM MK2206. Columns represent the percentage of the mean number of colonies versus control ± s.d.

We observed that *AKT1* silencing in MSTO-211H resulted in a significant decrease in total AKT phosphorylation (Figure 3A). To discriminate if the described phenotypic transition was due to the loss of AKT1 expression or to the loss of its phosphorylation/activation status, we treated MSTO-211H cells with MK2206, a highly selective pan-AKT allosteric inhibitor. Figure 3B shows a representative Western blot analysis that confirms the inhibition of AKT phosphorylation upon MK2206 treatment with no apparent effect on AKT levels. As shown in Figure 3C and 3D, the morphology in monolayer culture and *CDH1* expression did not change in response to treatment with MK2206 compared to control. In accordance, also network formation on Matrigel and number of colonies in soft agar were not affected by MK2206 treatment compared to untreated cells (Figure 3E, 3F).

### SIRT1 regulates AKT1 acetylation and protein interactions

To understand the functional role of SIRT1-mediated regulation of AKT expression and activation we analyzed the effect of depleting endogenous *SIRT1* in MSTO-211H cells.

**Figure 4:**
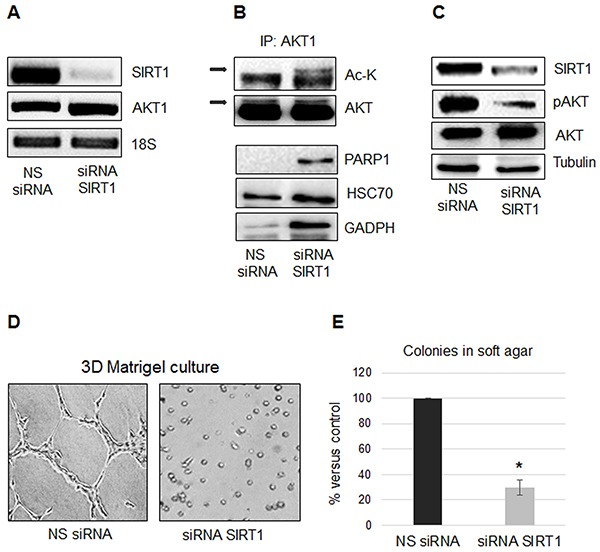
SIRT1 regulates AKT1 acetylation and protein interactions **A.** Representative RT-PCR analyses of *SIRT1* and *AKT1* in MSTO-211H cells transfected with non-specific control siRNA (NS siRNA) or *SIRT1* siRNA (siRNA SIRT1). 18S rRNA was used as housekeeping gene. **B.** Immunoprecipitation of AKT1, from lysates of MSTO-211H cells transfected with non-specific control siRNA (NS siRNA) or *SIRT1* siRNA (siRNA SIRT1); Lysine acetylation and co-immunoprecipitated proteins were detected by Western blot analyses using the respective antibodies (Ac-K, PARP1, HSC70 and GADPH). **C.** Representative Western blot analyses of SIRT1, pAKT and AKT in MSTO-211H cells transfected with non-specific control siRNA (NS siRNA) or *SIRT1* siRNA (siRNA SIRT1). Tubulin was used as loading control. **D.** Phase contrast images (200X magnification) of MSTO-211H cells transfected with non-specific control siRNA or specific *SIRT1* siRNA grown on Matrigel coated dishes for 24 hours. **E.** Soft agar colony counts in non-specific control siRNA or specific *SIRT1* siRNA transfected MSTO-211H cells. Columns represent the percentage of the mean number of colonies versus control ± s.d.; * p≤0.05.

As shown in Figure 4A, *SIRT1* silencing did not affect AKT1 expression, but increased its acetylation (Figure 4B) and inhibited its phosphorylation (Figure 4C). *SIRT1* silencing compromised the MSTO-211H cell spreading on Matrigel (Figure 4D) and significantly inhibited the number of colonies in soft agar (Figure 4E). We used co-immunoprecipitation coupled with MS/MS analysis to identify proteins that interacted with acetylated AKT1 following *SIRT1* silencing in MSTO-211H cells. The eleven proteins co-immunoprecipitated with acetylated AKT are listed in Table 1. As shown in Figure 4B, we confirmed the increased association of acetylated AKT1 with PARP1, HSC70 and GADPH.

**Table 1:** Proteins that interact with acetylated AKT1, identified by immunoprecipitation and mass spectrometry

Band	Protein identity	Data base accession number	Mr/pI
1	40S ribosomal protein S2	gi15055539	31305/10.5
2	glyceraldeyde-3-phosphate dehydrogenase	gi31645	36031/8.26
2	heterogeneous nuclear ribonucleoproteins A2/B1 isoform A2	gi4504447	35984/8.67
3	vimentin	gi62414289	53619/5.06
4	heat shock cognate 71kDa protein isoform 1	gi5729877	70854/5.37
5	alpha actinin 4	gi2804273	102204/5.27
6	hnRNP U protein	gi32358	88890/5.96
6	poly(ADP-ribose) polymerase	gi190167	113011/9.02
7	desmoplakin I	gi1147813	331571/6.44
7	filamin A	gi53791219	277332/5.7
7	actin-binding protein homolog ABP-278	gi3282771	278018/5.47

### AKT1/SIRT1/FOXM1 axis modulates ERβ expression

**Figure 5:**
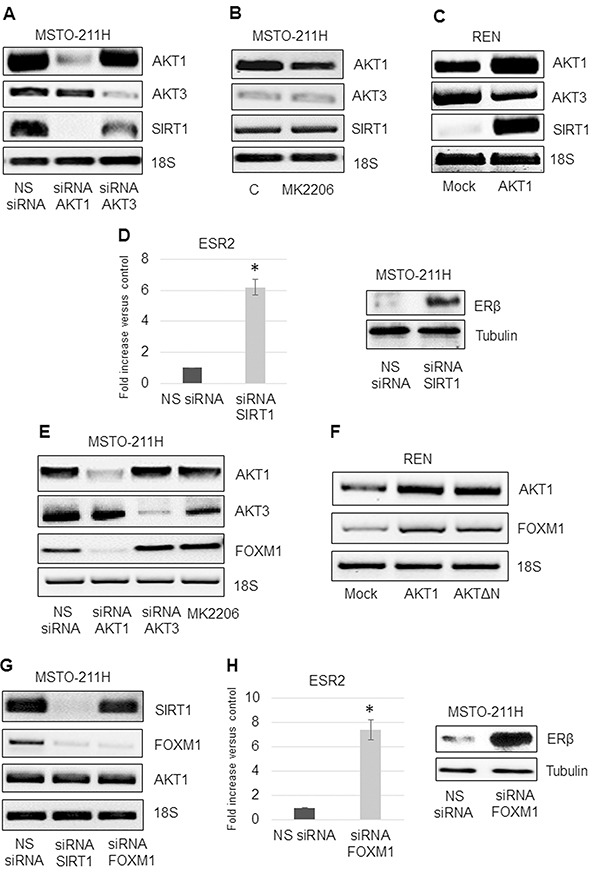
AKT1/SIRT1/FOXM1 axis modulates ERβ expression **A.** Representative RT-PCR analyses of *AKT1*, *AKT3* and *SIRT1* in MSTO-211H cells transfected with non-specific control siRNA (NS siRNA), and siRNAs of *AKT1* (siRNA AKT1) or *AKT3* (siRNA AKT3). **B.** MSTO-211H cells treated for 24 hours ± 8 nM MK2206. **C.** Mock or AKT1-HA transfected REN cells. 18S rRNA was used as housekeeping gene. **D.** Real time and representative Western blot analyses of ERβ expression in MSTO-211H cells transfected with non-specific control siRNA (NS siRNA) or *SIRT1* siRNA (siRNA SIRT1). Tubulin was used as loading control. **E.** Representative RT-PCR analyses of *AKT1, AKT3* and *FOXM1* in MSTO-211H cells transfected with non-specific control siRNA (NS siRNA), and siRNAs of *AKT1* (siRNA AKT1) or *AKT3* (siRNA AKT3) or treated for 24 hours ± 8 nM MK2206. **F.** Representative RT-PCR analyses of *AKT1* and *FOXM1* in Mock, AKT1-HA and AKTΔN transfected REN cells. 18S rRNA was used as housekeeping gene. **G.** Representative RT-PCR analyses of *SIRT1*, *FOXM1* and *AKT1* in MSTO-211H cells transfected with non-specific control siRNA (NS siRNA) or *SIRT1* siRNA (siRNA SIRT1) or *FOXM1* siRNA (siRNA FOXM1). 18S rRNA was used as housekeeping gene. **H.** Real time and representative Western blot analyses of ERβ expression in MSTO-211H cells transfected with non-specific control siRNA (NS siRNA) or *FOXM1* siRNA (siRNA FOXM1). Tubulin was used as loading control.

A positive feedback loop in which AKT1 and SIRT1 regulate each other's activity has been suggested [17]. We analyzed the expression of *SIRT1* in *AKT1* or *-3* silenced MSTO-211H cells by RT-PCR. As shown in Figure 5A *SIRT1* expression was dramatically decreased in *AKT1* but not in *AKT3* silenced cells. No significant effect on *SIRT1* expression was observed in MK2206 treated cells (Figure 5B), suggesting that inhibition of SIRT1 expression is not dependent on phosphorylated AKT1.

Conversely, *AKT1* overexpression in REN cells caused a significant increase in *SIRT1* expression (Figure 5C). In a recently published paper, we described that *AKT1* silencing resulted in increased expression of ERβ in the negative MSTO-211H cells [30]. In this study we show that also *SIRT1* silencing resulted in a similar increase in ERβ expression (Figure 5D). Given the described interplay between the transcription factor FOXM1 and SIRT1 [25], we decided to explore the role of FOXM1 in our system. We observed that *FOXM1* expression was inhibited in *AKT1* silenced but not in *AKT3* silenced or MK2206 treated MSTO-211H cells (Figure 5E). Overexpression of *AKT1-HA* or of the kinase-defective form of AKT (*AKTΔN)* resulted in increased FOXM1 mRNA levels in REN cells (Figure 5F). We established that in MSTO-211H cells, it was SIRT1 that modulated *FOXM1* expression and not vice versa (Figures 5G). The induction of ERβ in *FOXM1* silenced cells (Figure 5H) was indicative of a regulatory link between these two genes.

### The ERβ selective agonist KB9520 promotes AKT acetylation and protein interaction *in vitro* and *in vivo*

In our recently published paper, we described that in tumors from mice injected with MSTO-211H cells, *in vivo* treatment with the selective ERβ agonist KB9520 increased ERβ expression and AKT acetylation, due to decreased *SIRT1* expression [30].

**Figure 6:**
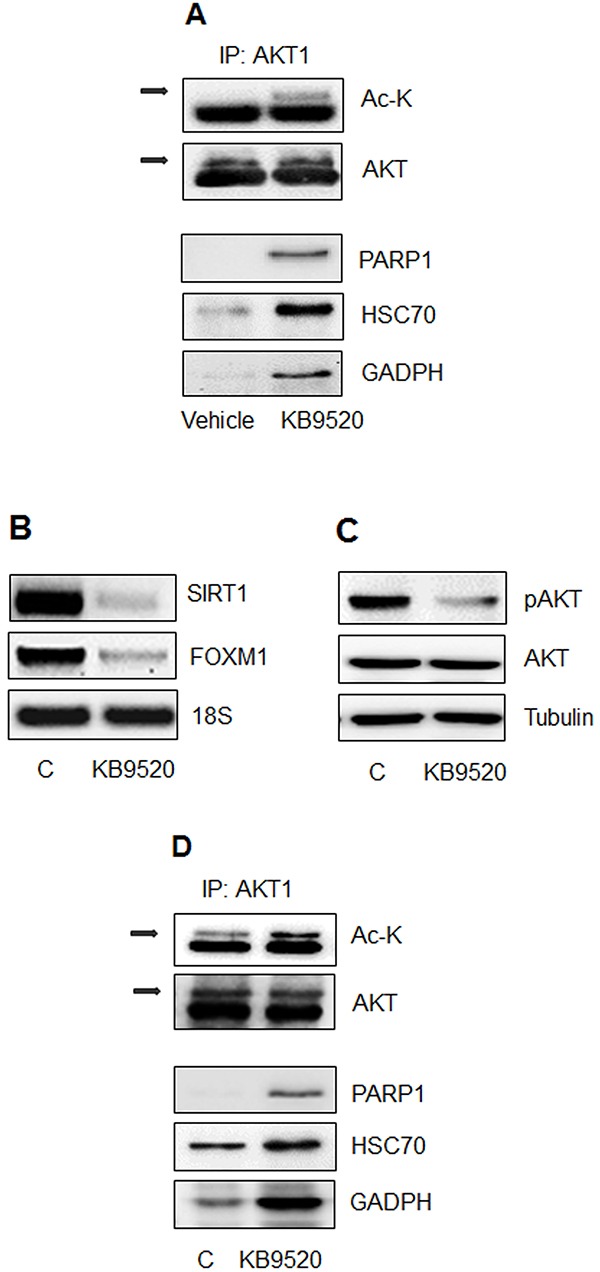
The ERβ selective agonist KB9520 promotes AKT acetylation and protein interaction *in vitro* and *in vivo* **A.** MSTO-211H tumors from mice treated for 25 days with vehicle or KB9520 (10 mg/kg/day); Lysine acetylation and co-immunoprecipitated proteins were detected by Western blot analyses using the respective antibodies (Ac-K, PARP1, HSC70, and GADPH). **B.** Representative RT-PCR analyses of *SIRT1* and *FOXM1* in REN cells treated for 24 hours ± 10nM KB9520. 18S rRNA was used as housekeeping gene. **C.** Representative Western blot analyses of pAKT and AKT in REN cells treated for 24 hours ± 10nM KB9520. Tubulin was used as loading control. **D.** Immunoprecipitation of AKT1, from lysates of REN cells treated for 24 hours ± 10nM KB9520; Lysine acetylation and co-immunoprecipitated proteins were detected by Western blot analyses using the respective antibodies (Ac-K, PARP1, HSC70, and GADPH).

Similar to *SIRT1* silenced MSTO-211H cells *in vitro* (Figure 4B), we observed an increased association of PARP1, HSC70 and GADPH with acetylated AKT1 in MSTO-211H tumors from mice treated with KB9520, compared to vehicle control animals (Figure 6A).

Likewise, in Figures 6B, 6C and 6D, we show that KB9520 treatment of ERβ positive REN cells *in vitro* caused decreased expression of *SIRT1* and *FOXM1* and a switch from phosphorylated to acetylated AKT1. The interaction of PARP1, HSC70 and GADPH with acetylated AKT1 in KB9520 treated REN cell is shown in Figure 6D.

## DISCUSSION

The AKT serine/threonine kinases are frequently active and play critical roles in the development and progression of various human cancers, acting on cell metabolism, survival, and proliferation [31, 32]. The role of AKTs in cell migration and metastases is less clear because of conflicting results, mainly depending on the cell and tumor type studied [33–37].

In this study, we show that AKT1 and -3 are expressed in MPM and that patients whose tumors express high levels of *AKT1* exhibit a significantly worse prognosis, while no significant correlation with *AKT3* expression is observed. We describe that *AKT1* isoform silencing in biphasic derived MPM cells causes a reversion from a spindle-like to a more epithelioid phenotype in monolayer culture, a reduction in spreading on Matrigel and a near complete inhibition of the 3D growth in soft agar. Moreover, data presented establish that these effects were independent of phosphorylated AKT1. *AKT3* silencing did not influence any of these processes. Mechanisms responsible for the distinct roles of AKT1 and -3 and the independence of pAKT1 to exert these effects are not known. However, different subcellular localization or binding partners may determine these isoform-specific functions and the pAKT independent effects.

Even though deregulation of PI3K and PTEN activity is a prevalent cause for AKT hyper activation in human cancers, other mechanisms have emerged. The discovery of posttranslational modifications such as acetylation, ubiquitination, SUMOylation and glycosylation adds further complexity to the regulatory networks controlling AKT signaling [17].

Members of the various classes of histone deacetylases (HDACs) have shown to be altered in different cancers and current views suggest that perturbed protein acetylation patterns may impact on tumor progression [38–40].

Our data demonstrate that *SIRT1* silencing increases AKT1 acetylation and suppresses aggressive properties of MPM cells. MS/MS analysis reveals that acetylated AKT1 interacts with proteins such as desmoplakin I, vimentin, alpha-actinin 4 and filamin, all known to be involved in intercellular junctions and cytoskeleton assembly. Furthermore, we confirm, by co-immunoprecipitation experiments, that acetylated AKT1 interacts with PARP1, HSC70 and GADPH. The role and the intracellular localization of acetylated AKT1, in complexes with these proteins, require additional studies.

SIRT1 has also been implicated in the modulation of the forkhead box protein M1 (FOXM1) [41]. Consistent with its role in cell proliferation, FOXM1 overexpression has been identified in many types of cancers, including liver, prostate, breast, lung, and colon [42]. This has been further confirmed by independent gene expression profiling studies of cancers, which identified *FOXM1* as a commonly up-regulated gene in human solid tumors [43].

**Figure 7:**
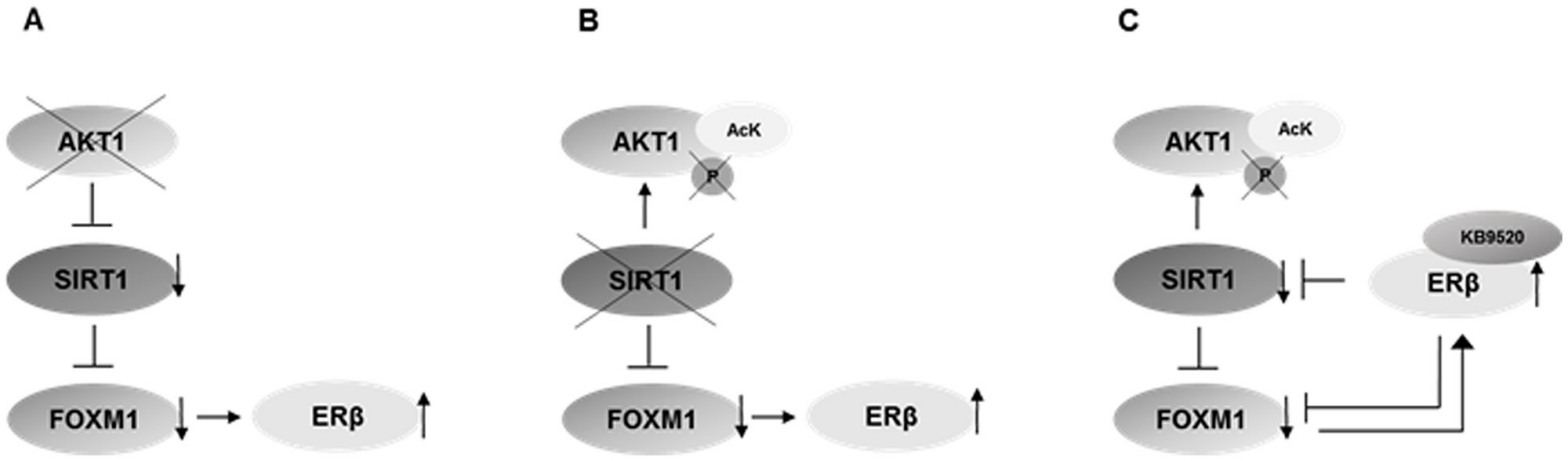
Schematic representation of the proposed mechanism **A.** Knockdown of *AKT1* inhibits the expression of *SIRT1* (Figure 5A) and *FOXM1* (Figure 5E), and increases ERβ expression [reference 30]. **B.** Knockdown of *SIRT1* increases the level of acetylated AKT1 (Figure 4B), decreases the level of phosphorylated AKT1 (Figure 4C), decreases the expression of *FOXM1* (Figure 5G) and increases ERβ expression (Figure 5D) **C.** Activation of ERβ with the selective agonist KB9520 inhibits *SIRT1* and *FOXM1* expression (Figure 6B), decreases levels of phosphorylated AKT (Figure 6C) and increases the level of acetylated AKT1 (Figure 6A, 6D).

Here we describe a role of the AKT1/SIRT1/FOXM1 axis in the regulated expression of the tumor suppressor ERβ, in MPM cells (Figure 7). Moreover, our data support a regulatory feedback loop exerted by ERβ on this axis. When ERβ is activated by the selective agonist KB9520, *SIRT1* and F*OXM1* are down regulated, resulting in increased acetylated AKT1 and interaction with PARP1, HSC70 and GADPH in tumor cells, both *in vitro* and *in vivo*. The observed ERβ mediated inhibition of *FOXM1* expression could occur via inhibition of AKT signaling through increased histone acetylation due to decreased *SIRT1* expression, and/or via a direct ERβ interaction with an ERE element located at −45 bp upstream of the transcriptional start site of *FOXM1*.

It has been reported that ERβ represses *FOXM1* expression in breast cancer cells primarily through competing with ERα on binding to the ERE element in the FOXM1 promoter [44]. Here we describe that ERβ negatively regulates the expression of the *FOXM1* oncogene in MPM cells, in the absence of ERα expression. The findings that activated ERβ is able to inhibit AKT1 signaling and *SIRT1* and *FOXM1* expression define a novel mechanism for the key anti-proliferative and pro-differentiating role of ERβ in MPM cells.

Despite the growing amount of research demonstrating the existence of AKT isoform-specific regulation, many papers still draw generalized conclusions about AKT function in cancer cells, without considering the unique function of each AKT isoform. In this report we demonstrate that inhibition of AKT1 phosphorylation by use of the allosteric AKT inhibitor, MK2206, is not sufficient to affect MPM cell aggressiveness. Moreover, our data reveal that AKT1 and -3 expression have different impact on cell behavior and gene expression. These results may aid in the development of targeted strategies for specific AKT isoform modulation in MPM therapy.

## MATERIALS AND METHODS

### Reagents and antibodies

The monoclonal antibodies specific for α-Tubulin, E-Cadherin, AKT1, PARP1, HSC70, GADPH and acetylated-lysine and the polyclonal antibodies specific for ERβ, SIRT1, phospho-AKT (pSer473), AKT, were purchased from Santa Cruz Biotechnology (Santa Cruz, CA, USA). Anti-AKT1 and AKT3 monoclonal antibodies were from Rockland Immunochemicals Inc. (Gilbertsville, PA, USA). Anti-mouse and anti-rabbit IgG peroxidase conjugated antibodies and chemical reagents were from Sigma-Aldrich (St Louis, MO, USA). ECL, nitrocellulose membranes and protein assay kit were from Bio-Rad (Hercules, CA, USA). Culture media, sera, antibiotics and LipofectAMINE transfection reagent were from Invitrogen (Carlsbad, CA, USA). The AKT-inhibitor MK-2206 was obtained from Selleck Chemicals (Houston, TX, USA). The, previously described ERβ selective agonist KB9520 [30, 45] was designed and synthesized by Karo Bio AB (Huddinge, Sweden).

### Cell cultures and transfection

The biphasic MSTO-211H cell line was obtained from the Istituto Scientifico Tumori (IST) Cell-bank, Genoa, Italy; the epithelioid REN cell line was isolated, characterized and kindly provided by Dr. Albelda S.M. (University of Pennsylvania, Philadelphia; PA, USA). Cells were grown in standard conditions in RPMI medium supplemented with 10% FBS, 100 μg/ml streptomycin and 10 μg/ml penicillin at 37°C in a humidified environment containing 5% CO_2_. Mycoplasma infection was excluded by the use of Mycoplasma PlusTM PCR Primer Set kit from Stratagene (La Jolla, CA, USA). Cells grown to 80% confluence in tissue culture dishes were transiently transfected with the pcDNA3 AKT1-HA #9021 plasmid, with the Flag-SIRT1 #1791 (Addgene, Cambridge, MA, USA) or with the plasmid encoding point mutant kinase negative HA-Akt (K179M) inpCMV6 (AktDN) (kind gift of T. Bobo, Columbia University, NY) using LipofectAMINE reagent as described by the manufacturer. Gene silencing was achieved by specific siRNAs from Qiagen (Hilden, Germany).

### *In vitro* adhesion to Matrigel

50 μl of Matrigel (Collaborative Biomedical Products, Bedford, MA, USA) were added to each well of a 96-well plate and allowed to form a gel for 30 minutes at 37°C. MSTO-211H or REN cells (1×10^4^ cells) in 100 μl of complete medium were subsequently added to each well and incubated 24 hours at 37°C, in 5% CO2. Under these conditions, cells form networks of tubes that are detectable within 2-4 hours and are fully developed after 24 hours.

### Assay for anchorage-independent cell growth

Anchorage-independent growth was determined using a modification of previously described methods [46]. Briefly, a base layer of 0.6% agar in complete medium was plated in six-well plates and allowed to solidify. Next, wells were overlaid with 5×10^3^ cells per well in a 0.3% agar. The plates were incubated at 37°C, in 5% CO2, and checked every 2 days for colony formation. At day 7, individual colonies (defined as clusters of 15 or more cells) were counted in 10 random fields.

### Cell lysis, immunoprecipitation and immunoblot

Cells were extracted with 1% NP-40 lysis buffer (1% NP-40, 150 mM NaCl, 50 mM Tris-HCl pH 8.5 mM EDTA, 10 mM NaF, 10 mM Na_4_P_2_O_7_, 0.4 mM Na_3_VO_4_) with freshly added protease inhibitors (10 μg/ml leupeptin, 4 μg/ml pepstatin and 0.1 Unit/ml aprotinin). Lysates were centrifuged at 13.000 x g for 10 minutes at 4°C and the supernatants were collected and as sayed for protein concentration with the Bio-Rad protein assay method. For immunoprecipitation experiments, 2 mg of extracted protein for each treatment were incubated with specific antibodies for 1 hour at 4°C and 50 μl protein A-Sepharose beads. Proteins were separated by SDS-PAGE under reducing conditions. Following SDS-PAGE, proteins were transferred to nitrocellulose, reacted with specific antibodies and then detected with peroxidase-conjugate secondary antibodies and chemioluminescent ECL reagent. Densitometric analysis was performed using the GS 250 Molecular Image (Bio- Rad).

### In-gel digestion

After co-immunoprecipitation experiments, bands 1-7 (30-300 kDa range) were excised from Comassie stained SDS-PAGE gel, cut into small pieces and destained with a solution of 50% methanol, 5% acetic acid. The gel pieces were shrunk with 100% ACN, dried in a SpeedVac, rehydrated with 100 mM NH_4_HCO_3_ for 15 minutes, then an equal volume of ACN was added for a 10 minutes incubation and the gel pieces were dried in a SpeedVac. Dried gel pieces were reduced with 10 mM DTT in 100mM NH_4_HCO_3_ for 30 minutes at room temperature, alkylated with 100 mM iodoacetamide in 100 mM NH_4_HCO_3_ for 30 minutes in the dark at room temperature. Digestion was performed overnight at 37°C with 25 ng μl^−1^ Trypsin in 50 mM NH_4_HCO_3_ (sequencing grade, Roche, Penzberg, Germany). Peptide extraction was carried out twice in 50% ACN/0,1% trifluoroacetic acid (TFA) for 10 minutes with ultra-sonication. The supernatants were pooled and lyophilized in a SpeedVac for mass spectrometry analysis.

### Protein identification by ESI-Q-TOF MS/MS analysis

MS/MS analysis was performed using a QSTAR XL hybrid quadrupole-TOF instrument (Applied Biosystems, Foster City, CA, USA) coupled with a LC Packings Ultimate 3000 nano-flow LC system (Dionex, Amsterdam, The Netherlands), as described by Bona et al. [47]. Briefly, the QSTAR XL operated in positive mode and in information-dependent acquisition (IDA) mode; the dynamic exclusion feature of the Analyst QS 1.1 software (Applied Biosystems, Foster City, CA, USA) was enabled, with an exclusion mass width of ± 3 m/z for 60 seconds. LC/MS–MS files obtained from each protein sample were merged into a single MASCOT generic format (mgf) file and searched against the NCBI non-redundant database; tolerance for precursor and fragment masses was 0.25 Da.

### RNA isolation and quantitative real-time PCR

Total RNA was extracted using the guanidinium thyocianate method. Starting from equal amounts of RNA, cDNA used as template for amplification in the real-time PCR (5 μg), was synthesized by the reverse transcription reaction using RevertAid Minus First Strand cDNA Synthesis Kit from Fermentas–Thermo Scientific (Burlington, ON, Canada), using random hexamers as primers, according to the manufacturer's instructions. The real-time reverse transcription–PCR (RT–PCR) was performed using the double- stranded DNA-binding dye SYBR Green PCR Master Mix (Fermentas–Thermo Scientific) on an ABI GeneAmp 7000 Sequence Detection System machine, as described by the manufacturer. The instrument, for each gene tested, obtained graphical Cycle threshold (Ct) values automatically. Triplicate reactions were performed for each marker and the melting curves were constructed using Dissociation Curves Software (Applied Biosystems, Foster City, CA, USA), to ensure that only a single product was amplified. The primers sequences are reported in Supplementary Table 1.

### *In vivo* experiments

#### Animals

CD1 nude mice (males, 6 weeks old; Charles River, Calco, Italy) received intra- peritoneal (i.p.) injections of 1×10^6^ luciferase transduced MSTO-211H cells in 0.5 mL of RPMI medium. An elapse of 15 days was allowed for the formation of detectable tumor nodules, assessed by IVIS® imaging. Mice were then weighed and stratified into treatment groups of ten animals. Treatment protocols were done from the 15th day to the 40th day, and mice were analyzed every 4-5 days by IVIS® imaging to assess tumor growth. One dose of KB9520 was used (10 mg/kg/day). KB9520 was dissolved in the vehicle (5% DMSO/40% PEG 400/55% water) and administrated once daily (days 15-40) by sub-cutaneous administration. Untreated animals were dosed with empty vehicle. At day 40 mice from the two groups were euthanized and necropsied. Tumors growing in the peritoneum were excised, and one part of the tumor tissues was immediately frozen and stored at −80°C for subsequent analysis. *In vivo* experiments were approved by Istituto Scientifico Tumori (Genoa, Italy) ethical committee and conform to the relevant regulatory standards. Mice were maintained and handled under aseptic conditions, and were allowed access to food and water ad libitum.

### Statistical analysis

Statistical evaluation of the differential analysis was performed by one way ANOVA and Student's t-test.

## SUPPLEMENTARY FIGURE AND TABLE


