# Supplementary material for: SIRT1 at the crossroads of AKT1 and ERβ in malignant pleural mesothelioma cells
Source: Oncotarget. 2016 Feb 11;7(12):14366–79. doi: 10.18632/oncotarget.7321 (PMC4924721; doi:10.18632/oncotarget.7321)
Supplement: Supplementary file 1 [file oncotarget-07-14366-s001.pdf]

SUPPLEMENTARY FIGURE AND TABLE

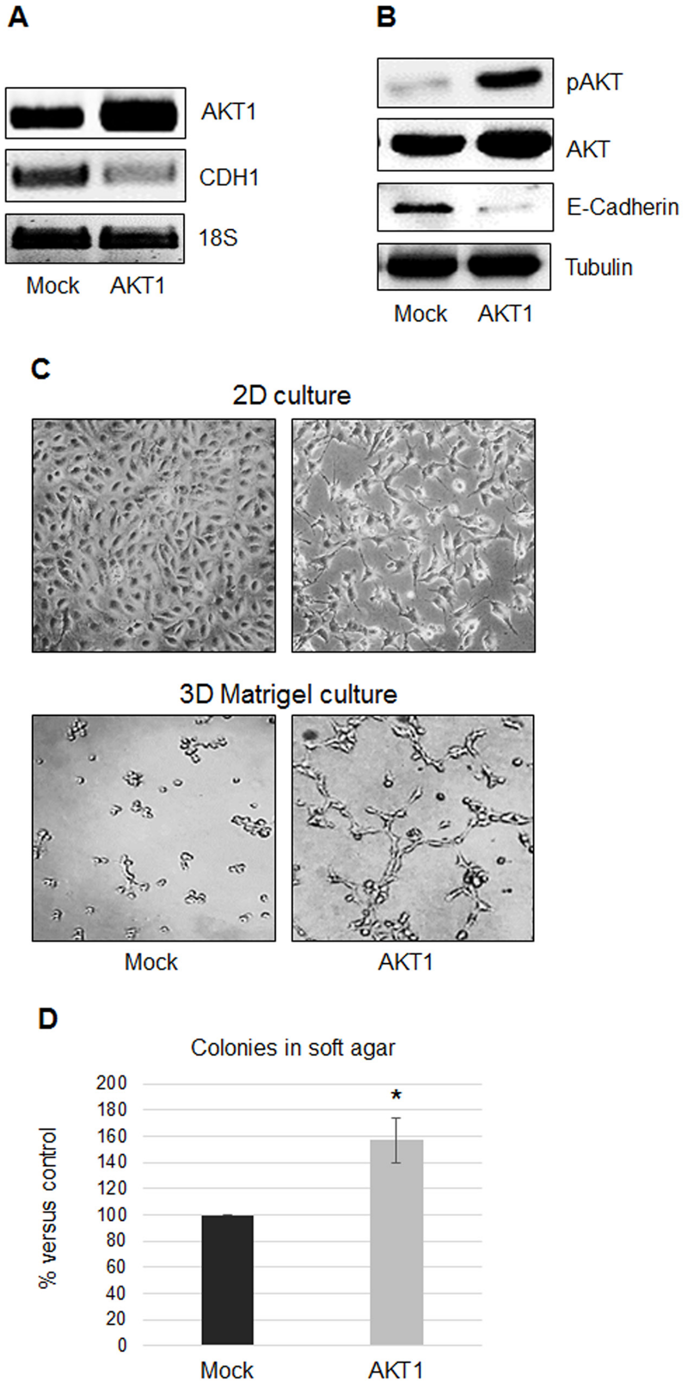

Supplementary Figure S1: AKT1 overexpression affects MPM cell morphology and anchorage-independent growth. **A.** Representative RT-PCR analyses of *AKT1* and *CDH1* in Mock or AKT1-HA transfected REN cells. 18S rRNA was used as housekeeping gene. **B.** Representative Western blot analyses of pAKT, AKT and E-Cadherin in Mock or AKT1-HA transfected REN cells. Tubulin was used as loading control. **C.** Phase contrast images (200X magnification) of Mock or AKT1-HA transfected REN cells grown on untreated plastic or Matrigel coated dishes for 24 hours. **D.** Soft agar colony counts in Mock or in AKT1-HA transfected REN cells. Columns represent the percentage of the mean number of colonies versus control ± s.d.; * $p \leq 0.05$.

Supplementary Table S1: Sequences of primers used for RT-PCR assays

GENE	PRIMER FORWARD	PRIMER REVERSE
18S	5′-AAA CGG CTA CCA CAT CCA AG-3′	5′-CCT CCA ATG GAT CCT CGT TA-3′
AKT1	5′- GCT GGA CGA TAG CTT GGA-3′	5′-GAT GAC AGA TAG CTG GTG-3′
AKT3	5′- GCA AGT GGA CGA GAA TAA GTC TC-3′	5′- ACA ATG GTG GGC TCA TGA CTT CC-3′
CDH1	5′-TGG GCT GGA CCG AGA GAG TT-3′	5′-ATC TCC AGC CAG TTG GCA GT-3′
SIRT1	5′-CTG GAC AAT TCC AGC CAT CT-3′	5′-GGG TGG CAA CTC TGA CAA AT-3′
FOXM1	5′-AAC CGC TAC TTG ACA TTG G-3′	5′-GCA GTG GCT TCA TCT TCC-3′
